# *Troglostrongylus brevior* in an Eurasian lynx (*Lynx lynx*) from Bosnia and Herzegovina

**DOI:** 10.1186/s13071-015-1272-9

**Published:** 2015-12-21

**Authors:** Amer Alić, Donato Traversa, Georg Gerhard Duscher, Mirsad Kadrić, Angela Di Cesare, Adnan Hodžić

**Affiliations:** Department of Pathology, Faculty of Veterinary Medicine, University of Sarajevo, Sarajevo, Bosnia and Herzegovina; Teaching Veterinary Hospital, Faculty of Veterinary Medicine, University of Teramo, Teramo, Italy; Institute of Parasitology, Department of Pathobiology, University of Veterinary Medicine Vienna, Veterinaerplatz 1, 1210 Vienna, Austria

**Keywords:** *Troglostrongylus brevior*, Eurasian lynx, *Lynx lynx*, Pneumonia, Bosnia and Herzegovina

## Abstract

**Background:**

In the past few years the interest of the scientific community on lungworms of the genus *Troglostrongylus* has grown due to the increased number of unexpected cases of infections with *Troglostrongylus brevior* in domestic cats from Mediterranean Europe, likely due to a spill-over from wild reservoirs. Thus, there is a merit to increase our knowledge on the occurrence of this parasite in felids from European regions. The present paper describes lung lesions associated with *T. brevior* infection in the endangered Eurasian lynx (*Lynx lynx*) from Bosnia and Herzegovina.

**Findings:**

The carcass of an illegally killed 3-year-old male Eurasian lynx was presented for necropsy at the Faculty of Veterinary Medicine of Sarajevo (Bosnia and Herzegovina). Grossly, multiple, multinodular, consolidated and firm, tan to grey areas, occupying the caudal third of caudal lung lobes, were observed. At cut section, the catarrhal fluid was draining from the airways. Larvae of *T. brevior* were found in tracheal scraping. The histopathological examination revealed multifocal to coalescing areas, centered on bronchi and bronchioles, and expanded alveoli filled with necrotic debris, degenerated inflammatory cells, mostly neutrophils and macrophages, and multiple cross sections of parasite larvae and thin-walled morulated eggs of lungworms. The paraffin-embedded lung samples were molecularly positive for *T. brevior*.

**Conclusion:**

This paper describes the first record of *T. brevior* in the Eurasian lynx and the associated host lung pathology. Given its pathogenic potential and the lack of data on troglostrongylosis in lynx populations, the occurrence and impact of *Troglostrongylus* spp. on wildlife health as well as the role of *L. lynx* as reservoir of infection for other felids, should be further investigated.

## Background

The genus *Troglostrongylus* Vevers, 1923 (Metastrongyloidea: Crenosomatidae) encompasses four species of parasitic nematodes that have been described for the first time from the respiratory system of wild felids, i.e. *Troglostrongylus troglostrongylus* Vevers, 1923, *T. brevior* Gerichter, 1949, *T. subcrenatus* Railliet and Henry, 1913 and *T. wilsoni* Stough, 1953 [1]. Animals become infected by ingesting the infective stages in the intermediate hosts, i.e. snails and slugs, or more frequently in paratenic hosts, i.e. amphibians, birds, reptiles and rodents [1, 2]. In addition, direct transmission of *T. brevior* from the queen to the kittens has recently been hypothesised [3].

Although regarded as a parasite of wild felids [1, 2, 4], in the past few years *T. brevior* has been described in domestic cats from islands of Spain [5], Italy [6, 7] and Greece [8] and from hilly and mountainous sub-Apennine areas of Italy [4, 9–12]. In these cases clinical presentation and lesions ranged from subclinical infections to severe pathologies with life-threatening or fatal outcomes in kittens and young cats [4–6, 8–12]. Furthermore, *T. brevior* may contribute to more severe lung pathology when co-infections with *A. abstrusus* occur [11]. In Italy, the endangered European wildcat (*Felis silvestris silvestris*) is the natural reservoir of *T. brevior* with prevalences of infection of up to 71.4 % [4, 8, 13–15]. A study carried out in southern Italy did not clarify whether this lungworm is a threat for wildcat populations, despite the high prevalence recorded and a negative correlation between *T. brevior* burden and body condition index [13]. Nonetheless, a more recent study has shown that *T. brevior* may actually cause moderate to severe lung lesions in infected *F. s. silvestris* [14].

Despite an apparent spreading of *T. brevior* in both domestic and wild cats [4, 13–15], no reports of infections with *Troglostrongylus* spp. in another endangered European wild felid, i.e. the Eurasian lynx (*Lynx lynx*), are available. Indeed, wild *Lynx* spp. are frequently infected with *T. wilsoni*, i.e. the bobcat (*Lynx rufus*) in several regions of the United States [16, 17] and the Canada lynx (*Felis canadensis*) in northern Ontario [18]. Given the merit to enhance our knowledge on the occurrence and the impact of troglostrongylosis in the European wildlife, this paper describes for the first time pneumonia associated with *T. brevior* in an Eurasian lynx illegally killed in Bosnia and Herzegovina.

## Methods

In February 2014, the carcass of a *c.* 3-year-old male Eurasian lynx was presented for necropsy at the Department of Pathology, Faculty of Veterinary Medicine in Sarajevo. The lynx was shot by poachers in Central Bosnia, in the Fojnica municipality (43°57'34"N, 17°54'10"E) on the eastern slopes of the Vranica Mountain. The animal was in a good body condition. Tissue samples from lungs, kidneys, intestine, stomach, liver and spleen, were fixed in 10 % neutral buffered formalin overnight, embedded in paraffin, and cut at 3 to 6 μm sections. Deparaffinised sections were stained with hematoxilin and eosin and examined under a light microscope. Tracheal mucosa was scraped and examined under the light microscope. Genomic DNA was extracted from three paraffin-embedded lung tissue samples where morulated eggs and embedded nematode first stage larvae were analysed as previously described [19], molecularly examined [20] and the amplicon sequenced. The sequence obtained was aligned using Data Analysis in Molecular Biology and Evolution version 4.5.55 (DAMBE) and compared with those of other nematodes available in the GenBank® using the Nucleotide-Nucleotide “Basic Local Alignment Search Tool” (BLAST).

## Results

**Fig. 1:**
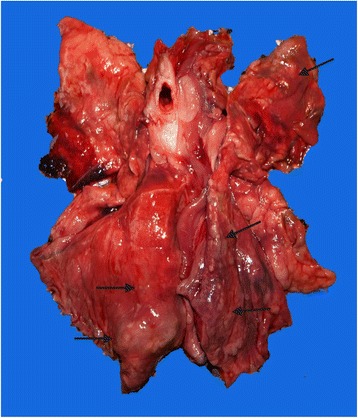
Multifocal, various sizes grey to tan consolidated areas (*arrows*) in the parenchyma of the caudal lung lobes of the Eurasian lynx infected with *Troglostrongylus brevior*

**Fig. 2:**
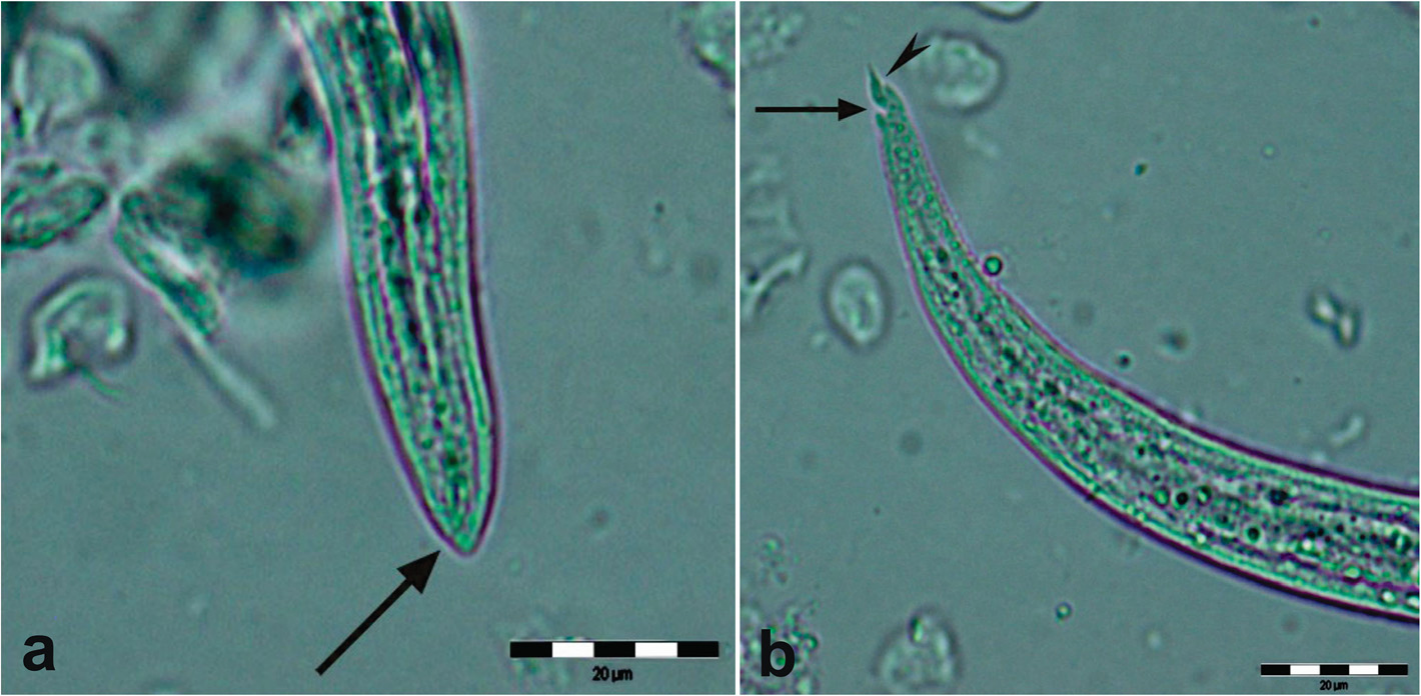
First stage larva (L1) of *Troglostrongylus brevior.* **a** Anterior end with a subterminal opening (*arrow*). **b** Pointed tail with a pronounced dorsal (*arrow*) and shalow ventral (*arrowhead*) incisures and spines typical of *T. brevior. Scale-bars*: 20 μm

At necropsy, multifocal, multinodular, consolidated and firm, tan to grey, areas, occupying the caudal third of both caudal lung lobes were observed (Fig. 1). Multiple 0.5 to 1.5 cm foci of similar appearance were present on the apical and cardiac lobes. Lung parenchyma was bright-red and a focal perforation with haemorrhage and oedema (bullet wound) was recorded on the caudal acute edge of the left apical lobe. At cut section of the lung, catarrhal fluid was draining from the airways. The tracheal mucosa was covered with a thin layer of red tinged (with blood) mucous material. In the scrapings of the tracheal mucosa multiple first stage larvae (L1) of *T. brevior* were detected (Fig. 2). No adult parasites were found. No remarkable lesions were observed on other organs.

**Fig. 3:**
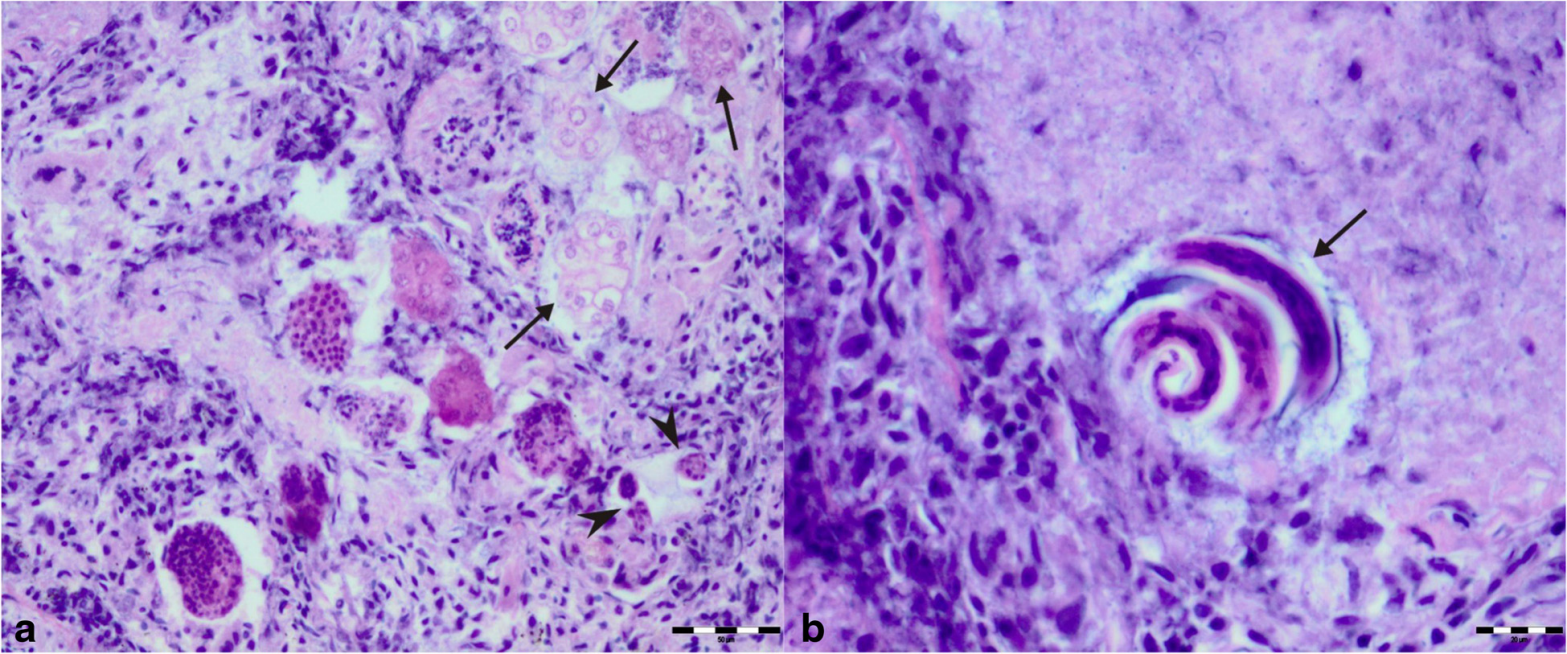
Histopathology of lung lesions associated with *Troglostrongylus brevior* infection in an Eurasian lynx (*Lynx lynx*). **a** Distended alveolar ducts and bronchioles filled with exudate, degenerated cells and multiple cross sections of parasites (*arrowheads*) and thin walled morulated eggs (*arrows*) in the lung of Eurasian lynx infected with *Troglostrongylus brevior*. **b** First stage larva (L1) (*arrow*) of *Troglostrongylus brevior* in the exudate filled distended alveolar duct of the Eurasian lynx. Hematoxilin and eosin staining. *Scale-bars*: **a**, 50 μm; **b**, 20 μm

Histopathology of lung tissue revealed multifocal to coalescing areas, centred on bronchi and bronchioles, and expanded alveoli filled with necrotic debris, degenerated inflammatory cells, mostly neutrophils and macrophages, and multiple cross sections of parasite larvae and thin walled morulated eggs (Fig. 3). The inter-alveolar septa were necrotic and expanded with moderate infiltrates of neutrophils, macrophages and lesser numbers of eosinophils. At the periphery of these areas, small numbers of multinucleate giant cells were observed. The walls of airways were expanded with moderate infiltrates of mostly neutrophils and eosinophils, and oedema. Multifocal necrotic areas filled with numerous neutrophils and lesser numbers of macrophages and eosinophils were scattered across the remaining parenchyma. There was a mild perivascular and peribronchiolar oedema, mild hyperplasia of media of vessel walls, and multifocal moderate perivascular cuffing of lymphocytes and neutrophils. Homogenous eosinophilic granular masses (thrombosis) were present in the lumen of multiple blood vessels. The paraffin-embedded samples scored molecularly positive for *T. brevior*, with a 100 % homology with the GenBank® sequence KF241978.1.

## Discussion

The data provided here represent the first report on the occurrence of *T. brevior* and associated lung pathology in the Eurasian lynx, *L. lynx*. Interestingly, until now *Troglostrongylus* spp. has not been detected in several studies of the Eurasian lynx parasite fauna in Europe [21–24], despite these lungworms occur frequently in bobcat and Canada lynx in North America [16–18]. The absence of *Troglostrongylus* spp. in the European lynx could be related to the use of only copromicroscopic and morphological methods applied in the previous studies, or due to the geographic area of sampling, that could be unsuitable for the biology of this lungworm [21–24]. Thus, the infection might have been misdiagnosed for the cat lungworm *Aelurostrongylus abstrusus* Railliet, 1898 [25]. In fact, *L. lynx* was considered a host of *A. abstrusus* [25], based on past copromicroscopic findings [26]. Nevertheless, morphometric data of those L1 identified as *A. abstrusus* [26] do not fall within the ranges reported for this nematode [6] and could represent a misdiagnosis of a *Troglostrongylus* spp. [27].

The lesions observed in the lung of the present case are almost identical to those described in a kitten co-infected with *A. abstrusus* and *T. brevior* [11]. No adult parasites were observed in the trachea or the bronchi of this lynx. The presence of larvae and morulated eggs in distended alveolar spaces surrounded with eosinophils, neutrophils and macrophages are lesions more attributed to *A. abstrusus* than to *T. brevior.* Lesions caused by *T. brevior* and *A. abstrusus* differ in localisation and intensity, as *T. brevior* is larger and mostly localised in the medium-sized and large bronchi and bronchioles, whereas *A. abstrusus* is smaller and localised in the alveolar ducts and subpleural parenchyma [1]. In cases of mixed infections the overlapping of lesions makes challenging to discern the exact cause of lung damage [11]. A mixed infection by *A. abstrusus* and *T. brevior* can be here considered unlikely because of the lack of *A. abstrusus*-typical lesions (e.g. subpleural nodules) and the negative result for *A. abstrusus* in a very sensitive DNA-based assay [20]. Additionally, a catarrhal bronchitis, as here described, is typical of troglostrongylosis in both domestic [1, 10, 11] and wild cats [14], and less evident in aelurostrongylosis, where macrophages are often organised in granulomas [28]. In the present case, however, no subpleural nodular aggregates of macrophages were observed. Furthermore, L1s (Fig. 2) detected in the tracheal scrapings have the typical characteristics of *T. brevior*. In any case, the presence of *A. abstrusus* in the animal examined here cannot be ultimately ruled out because the genomic DNA of *A. abstrusus* obtained from parrafin-embedded tissue could have been too fragmented to provide binding sites from specific primers or because *A. abstrusus* was not present in the three examined samples.

No lesions caused by *T. wilsoni* have been described in *Lynx* spp., apart from the proteinaceous fluid noted in the lung of one bobcat in Alabama, United States [17]. Although the cause of death of the lynx of the present case was a disgraceful and illegal killing by poachers, the extent of the recorded pneumonia demonstrates the ability of *T. brevior* to cause lesions and to hamper the respiratory system of this wild felid. Hence, further studies are warranted to investigate the occurrence of lungworms in this endangered felid species and if and how they may represent a threat for host's health and welfare. In fact, as the domestic cat, the Eurasian lynx could be affected by an overflow of pathogenic lungworms that are usually harboured by wildcats [4].

## Conclusions

This paper describes for the first time infection with *T. brevior* in an European lynx and the associated lung pathology. Given its pathogenic potential and the lack of data on *Troglostrongylus* spp. in lynx populations, the occurrence and impact of lungworms on Eurasian lynx health and welfare should be further investigated. Moreover, further studies are necessary to better elucidate the epizootiology of *T. brevior* in felids other than *Felis* spp., in order to understand whether the infection in the European lynx is due to bridging infections [2, 4] or, as indicated for the European wildcat [2, 4, 13], it represents another reservoir of troglostrongylosis.
